# Hospital School Program: The Right to Education for Long-Term Care Children

**DOI:** 10.3390/ijerph182111435

**Published:** 2021-10-30

**Authors:** Giuseppina Caggiano, Lucia Ilaria Giulia Brunetti, Kathleen Ho, Angiola Piovani, Alessia Quaranta

**Affiliations:** 1 Department of Biomedical Science and Human Oncology, School of Medicine, University of Bari Aldo Moro, 70125 Bari, Italy; alessia.quaranta@uniba.it; 2 Department of Clinical and Experimental Medicine, School of Medicine, University of Foggia, 71121 Foggia, Italy; luciabrunetti93@libero.it; 3 Hospital School, Lucile Packard Children’s Hospital at Stanford, Stanford Children’s Health, Palo Alto, CA 94304, USA; kho@stanfordchildrens.org; 4 Monte San Michele School, Giovanni XXIII Children’s Hospital, 70125 Bari, Italy; angiola.piovani@posta.istruzione.it

**Keywords:** hospital school program, hospital school teacher, hospitalization in children, school failure, school drop-out

## Abstract

Education and health are two inseparable aspects of a single dynamic which aims to support and increase the physical and mental well-being of children and young people. Children must be guaranteed two rights: the right to study and the right to health. Schools capable of reconciling these two fundamental needs are represented by school in hospital and home schooling. Thanks to this flexible teaching method, it is possible to support the child and his or her family during hospitalization, and to prevent consequences such as school failure and dropout. Hospitalization is always a traumatic event for children, in which white coats are unknown figures, perceived all the more threatening the younger the child: a threat to one’s integrity, loss of autonomy, distorted perception of time, loss of confidence, and a sense of abandonment. Therefore, it is important to create a communicative basis that facilitates the child’s adaptation to the new hospital environment and establishes continuity during this period of time. Teachers play a significant role within the context of such difficulties. They need to understand patients’ emotions and act as a bridge between the small inpatient room of the child and the outside world. In this article we examined: (1) the School in Hospital and the reasons why it is a valid resource for the psychophysical rehabilitation of the student in a hospital; (2) the role of the teacher in hospital and the difficult context in which the teacher has to work; and (3) how the school in hospital was challenged by the SARS-CoV2 pandemic.

## 1. Introduction

### The Hospital School Program over the Years

Hospital School is a tool that can guarantee the right to study for children and adolescents with chronic disabilities and/or illness, and for which frequent treatments and prolonged hospital stay are required. Born out of the need to offer hospitalized patients the same opportunities as their peers to acquire skills and knowledge, the Hospital School is now recognized as a part of the therapeutic program in the medical field.

The issue of the inclusion of “pupils affected by pathologies” in society has been configured over the years as an increasingly urgent question, especially in this period of global pandemic. To be addressed, this question needs to be “thought about”; that is, an approach must be intentionally designed. In fact, it is not possible to imagine that such paths of inclusion are the result of chance or fortuitous circumstances. In contrast, such a prospect of social integration requires intentional and conscious promotion of opportunities for inclusion in society. 

The Hospital School Program was born in Italy in the 1950s when some special school branches opened in pediatric wards. The purpose of the Hospital School Program was to support young patients in avoiding the typical difficulties and problems of going back to their original schools, thanks to the educational and learning support of primary school teachers. It is a tool that guarantees the right to study for children and adolescents suffering from chronic diseases that are often disabling and for which long-term care with prolonged hospitalization or stay at home is required.

Until the middle of the last century, the importance of addressing the needs of the whole-child was not adequately assessed in the pediatric setting. During long term hospitalization, the attention was focused exclusively on the management of the disease, neglecting the psychoemotional sphere of the pediatric patient. The child was removed from his environment and family, believing that he could be more calm without the constant presence of his parents. Contrarily, this often generated states of depression, aggression, and internal conflicts in the child, inducing changes in the development of his personality. It was Anna Freud, with research that began in the 1930s, who first probed the world of the sick child and demonstrated the enormous difference between the unconscious experiences and fantasies of adults versus those of children towards the disease. The main scientific evidence that emerged from the descriptions mothers made of their children’s behaviors during or after illness and hospitalization included severe mood changes, changes in relationships with parents and siblings, onset of eating and sleep disorders, onset of apathy, enuresis, regressions or, on the contrary, pathological accelerations in behavioral maturation [1]. In 1959 in England, a group of doctors and psychologists, coordinated by Sir Harry Platt, began to investigate the non-medical aspects of hospitalization for children in the UK. They pointed out, through the drafting of a document (“The Welfare of Children in Hospital”, better known as “Platt Report”), that the separation from the family caused malaise and behavioral disturbances [2]. To reduce this state of discomfort, they provided some solution suggestions: eliminate the limit of visiting hours, allow parents to stay overnight with the child, create spaces for play. It was therefore only starting from the 1960s, in the wake of publications such as “Hospitals and children” (a milestone in the complaint against the neglect of scientific and political institutions towards the needs of hospitalized minors) and “The Platt Report”, that Volunteer Associations in various European countries began to appear. These groups worked with support institutions and operators in promoting hospital “humanization” initiatives in order to share with families the management of supporting the patient. The awareness that the emotional sphere of the child could not be ignored, and the need to implement those interventions aimed to protect his or her psychoemotional well-being, began to spread in several European countries. This cultural shift occurred at the same time the World Health Organization (WHO) extended the concept of “health”, considering it no longer as “*the absence of disease or infirmity, but as a state of complete physical, psychological and social well-being*”.

The need to build a child-friendly hospital became essential, as well as promoting a process of humanization of the health contexts. From this time on, school branches in children’s hospitals increased to provide educational, emotional, and psychological support to ill children and their families. 

In Italy, a series of organizational and structural directives were drawn up on this matter in the early 1980s. The Ministerial Directive (DM) n. 345 of 2 December 1986 validated the training of school branches in hospitals to ensure a legitimate learning program, recognizing them as new locations in the territorial school district [3,4].

The subsequent Ministerial Directive (DM) n. 353 issued in 1998 confirmed that the Hospital School Program recognized the right and duty to education and acknowledged the risk of possible school dropout [5]. This particular educational aspect is only part of a larger project and strategy to prevent early school leaving. 

Nowadays this service can boast the collaboration of numerous teachers of all levels to provide a satisfactory level of education to both hospitalized and home students. The school service in hospital is a service in all respects offered by some schools of various degree and is not a service on a voluntary basis; it refers to a precise regulatory framework, including, in particular, the DM n. 353/1998, that regularized the school in hospital and the many sections operating in hospitals. The school in hospital is accessed for personal availability, once the teacher is included in the staff of schools with hospital section.

If tenured teachers need to apply for transfer to the institution which also has the school section in hospital, it is the school manager who, as for the other classes of the institute, makes the assignment to the section in hospital in compliance with the general criteria established by the collegial bodies.

The Hospital School Program is a national flagship system in education. This service has been up and operating throughout the national area. In Italy, for the academic year 2019–2020, 41,367 students took advantage of this service, mainly kindergarten and primary school students (about 70%) and 4250 high school students, with the collaboration of 915 teachers. For the academic year 2017–2018 in Apulia, the Hospital School Program engaged 1377 teachers: 58% were primary school teachers, 19% kindergarten teachers, 11% secondary school teachers, and 12% high school teachers [4]. 

The main goal of the Hospital School Program is to mitigate the crushing physical and mental impact on hospitalized patients and their families. This initiative mainly focuses on giving hospitalized students equal opportunities compared to their peers and preserving educational and existential continuity until they return to school, as enshrined in Article 14 of the Charter of Fundamental Rights of the European Union: “Every child has the right to education, whatever his condition” [6].

The Hospital School Program is part of a broad therapeutic process that aims to overcome the fear once hospitalization ended, in order to prevent the school drop out risk.

The smooth transition back to school after hospitalization or an extended period at home due to chronic illness is a crucial factor for preventing the onset of psychopathological disorders which in many cases may lead to the risk of early school leaving [7,8]. The School in Hospital (Si-Ho) also acts as a support for suffering parents thanks to the close presence of a teacher as a way to keep parental anxieties under control and not feel so alone in an everyday life completely subverted by disease and hospitalization [9].

## 2. A Complex Hospital Setting and the Complicated Role of Si-Ho Teacher

Hospitalization is always a traumatic event for children, in which white coats are unknown figures, perceived all the more threatening the younger the child. If the disease causes anxiety in the child, hospitalization is experienced as an uncontrollable threat to his or her integrity via loss of autonomy, distorted perception of time, loss of confidence, and a sense of abandonment. Furthermore, hospitalization is a stressful event that causes the alteration and disruption of the usual habits of life. Going to school, together with maintaining social relations with peers, and practicing activities and sports is certainly one of the cornerstones around which the life of a child takes place. Consequently, a pediatric hospitalized patient will inevitably perceive the lack of the school environment, in terms of educational activities, contact with teachers, and friendship relationships with their peers. Some of these children will have a specific anxiety to fall behind tasks in comparison with their classmates, with consequent negative effects on their school performance [10]. 

Literature has mentioned that hospitalized youngsters are more likely to experience learning difficulties, which may compromise their academic motivation and entail emotional struggles (e.g., higher risk of psychosocial problems). Additionally, hospital stays may increase the risk of students disengaging from school, e.g., mostly due to absence from school. This is particularly important, as school disengagement may lead to early school dropout, educational underachievement, or academic failure, e.g., a lower likelihood of completing compulsory education or entering university. Lastly, hospitalization may occur during critical transition points, such as starting school or key stages of learning and development, such as the onset of adolescence [11]. Therefore, it is important to create a communicative basis that facilitates the child’s adaptation to his new environment and establishes continuity in the change. Teachers play a very significant role within the context of such difficulties. They need to understand patients’ emotions, acting as a bridge between the small inpatient room of the children and the outside world. Furthermore, teachers are able sustain and stimulate interest in the hospitalized pupil; when a child is interested in something, he or she “forgets” the disease and overcomes boredom and hospital depression, especially in long-term care. In doing so, Si-Ho teachers become the guardian of student interests and the catalyst of their hopes for the future. Although improvements have been made to change the children’s hospital into a more human and inclusive place for young patients, the hospital setting still remains a dysfunctional place to host learning programs. Teachers assert that the causes responsible for this failure are mainly the inappropriateness and the lack of dedicated areas, the lack of teaching aids, and the priority of clinical activity over the didactic one, which is constantly interrupted [2,12].

Moreover, there is a sense of marginality reported by teachers who should be actively involved in the therapeutic process established for children joined to their families. They report to feel like “strangers whose presence is tolerated” [12,13].

On the contrary, an element that the teacher found gratifying is the possibility that the work carried out in the hospital context might give the students and their families a sense of continuity to their normal lives [14]. 

Therefore, a teacher should have certain personal characteristics along with educational and technological ones for effective action.

There are several key features necessary for this role. First, it is important to be able to adapt to rescheduled programs and objectives, depending on the number of patients, the different levels of learning, the severity of the symptoms they have, and the length of hospitalization. Given the complexity of this educational scenario, teachers opt for an individualized approach and sometimes conduct activities in small groups [15]. Unlike what happens in the classroom, the teacher who works in the hospital tends to modulate the educational proposal on the flows of the complex cognitive-emotional needs of the hospitalized pupil, and to stimulate learning with a view to personalization. In fact, rather than pursuing pre-determined and standard cognitive goals, the teacher sets up, together with the pupil, paths to promote all aspects of the child and his overall well-being. These paths may see the teacher and Hospital School as a “training agency”, protagonist of the “mediation” between being a child and the reality of the hospital environment in which he lives. Hospital teachers should then organize their teaching activity around the students’ state of physical health and psychological wellbeing, as well as needs triggered by the experience of illness and isolation. An important prerequisite for the effectiveness of teaching is surely planning; from a praxis point of view the teachers will:

- Establish how many children are present each day in hospital and/or are in physical condition to be able to take part in learning activities.
- Introduce the hospital school to newly admitted young patients and their families.
- Establish contact with the newly hospitalized students’ mainstream school teachers to acquire information on his or her skill levels.
- Evaluate the type and the length of the hospitalization.
- Design and implement a learning pathway for the student.
- Send the students portfolio containing his or her work and results during time in hospital to mainstream school at the end of the hospitalization [14].

Furthermore, research identified the need to develop educational programs to the maintenance of the relationships of the inpatient with their peers, and promote students’ motivation, Self Regulation Learning (SRL) strategies, and school engagement. According to Azvedo [11], the SRL might be an important resource for the students. It comprises some phases such as Planning, Execution, and Evaluation.

The Planning phase precedes the performance of the task and refers to the moment when it is expected that the students define their goals and select learning strategies to help them achieve their goals. In the Execution phase, the pre-established plan is implemented and monitored. Lastly, the Evaluation phase involves the analysis of the achieved results accounting for the established goals. In this way, students assume an active role in their own learning process and control over their educational paths, especially when facing difficulties. Training in SRL strategies provides students with necessary skills to influence their own cognitive and behavioral functioning. In fact, students who use self regulation strategies control their cognition, motivation, learning environments, and behavior throughout cognitive and metacognitive processes.

Two essential skills in this niche teaching work concern the ability to problem-solve (and teaching in the hospital poses new and unexpected problems to the teacher on a daily basis) and effective communication skills. 

The teacher is involved in a careful and constant work of receiving, decoding, and responding to the signals—even if not explicit, but equally significant—emitted by the sick child. This requires an intentional attitude of “listening” and of authentic interaction with the sick child. Additionally, there is the constant need to identify and overcome any communication disorders, mainly of a “contextual”, psychological, or physiological type, related the state of restlessness or fear of the hospitalized child, his or her feelings of inadequacy or inconstancy, desire for distraction, or need for solitude. Such “interference”, if not exactly identified and overcome, can contribute to communication failure with the hospitalized child. In short, a required empathy is needed to get in deep touch with the pains and sufferings of patients and their families, promoting a mutually confident relationship. 

Another aspect of the complexity of the hospital situation concerns the relationship with parents who, together with the medical team, dictate the modalities and regulate the rhythms of life of their sick child. Thanks to the communicative skills of the teachers, it is possible to establish harmony in relationships and create the serene atmosphere necessary for the child to overcome the disruption of their previous life, and to perceive the reality of the hospital only as a phase of transition. As school is a part of every child’s day-to-day life, interactions with Si-Ho teachers and the expectation that a child’s education will continue brings a sense of normalization to the traumatic world of the hospital setting.

Finally, it is crucial to deal with the lack of dedicated areas, lack of teaching aids, and constant interruptions from teaching activity. According to the study, all these aspects represent stress indicators factors for teachers in hospitals [14].

A protective factor for the susceptibility and risk of Si-Ho teacher burnout and emotional overload is a strong orientation towards teamwork skills. Sharing methods with both colleagues who teach in hospitals and with those who teach in local schools can lead to a unique and resilient network. Although challenged by the previously mentioned stressors, resilient teachers find gratification in the academic achievements of their students. In fact, although the Si-Ho Programs put teachers in an atypical professional condition which might expose them to the risk of worsening their psycho-physical state, they can also represent a particularly rewarding context that acts as a positive catalyst influencing teachers’ attitude and performance [16].

## 3. How Did the Hospital School Program Change during the SARS-CoV-2 Pandemic?

As a result of the SARS-CoV-2 pandemic, the hospital school program experienced a setback in the same way as the territorial school. Children’s hospitals have often turned into “COVID red zone” areas to the detriment of non-emergency wards. Hospitals with emergency therapy units only, in fact, reduced the days of hospitalization to a minimum, leading to severe problems in the relationship with the patient/student. Children with pathologies that require several hospitalizations have had the worst of it. Under normal conditions, they know that hospitalization does not imply interruption of teaching activities but having suspended or modified some of their therapeutic treatments they had to suspend the relationship with the SI-HO too.

The teachers felt confused and scared, and it took weeks to reorganize the hospital school curriculum through distance learning. This organization was not without its difficulties. The teachers expressed the complications in the first approach to the young patients and their families, without the active involvement of the medical staff. It was also difficult to access Hospital School Program student records for privacy reasons.

Some authors showed how difficult it was to achieve and maintain a humane and supportive long-distance relationship over time, especially with multi-ethnic families or those with poor technological skills [17]. After all, it is indisputable that direct, participatory teacher–student interaction is irreplaceable for the promotion of the child’s psycho-affective resources: with the face-to-face relationship, the emotional cues, individual and contextual variables, and interpretation of explicit messages can all be fostered in real time and in real space. However, it cannot be denied that new technologies and the potential of the virtual learning networks, applied to the School in Hospital, can allow for new opportunities and training courses aimed at the well-being of the sick pupil. In fact, students were equipped with technological devices; paper material was occasionally printed and delivered to patients in the “red zones” when necessary. In this way, the inconvenience of missing educational activities organized by school was largely reduced. 

Recently, thanks to the increasing affirmation of new technologies and their implementation in common daily life, it was thought to use technologies to improve the School in Hospital [18]. In particular, technology may help to overcome the obstacle created by the COVID-19 pandemic that forced Si-Ho teachers outside the hospital and away from direct patient interaction. This is the case of Dovì, an experimental project coordinated by Regional School in Molise (Italy) [19]. 

Dovì is a robot, equipped with interactive multimedia whiteboards and small drones that allow for a connection between the student in a hospital setting or at home with their schoolmates in a traditional classroom. The project aims to break down the physical and moral barriers that still hinder the right to study. In this way the school is no longer a physical learning place but an available virtual space able to include all the students with their experiences, promoting the exchange between them. 

During the pandemic, it can be noted that children with pathology have evened out with their classmates. Teachers of all schools connected via the web with all the pupils in the same way, both pathological and healthy ones, indistinctively. However, this approach led to poor results. Such standardization generated problems, as only Si-Ho teachers have “know how” requisites to cope with hospitalized pupils. Si-Ho students frequently suffer from loss of concentration due to the drugs taken for the therapy. For this reason, it is recommended that teaching is tailored to the needs of each individual, taking into account the age and the pathology they are suffering from. Through cooperation, school becomes a community, it becomes a family. It is a community care. It is known that an educational project works more effectively when the different agents collaborate towards a broader goal, in a sort of orchestration of a path. School in Hospital teaching aims for the complete recovery of the patent/student at all levels; the didactic intervention must be harmonized with the ongoing educational process. 

## 4. Conclusions

### Hospital School Program: Finding Opportunities

It is important to consider the child a “global being” in which cognitive, affective, and motor development influence each other in a mutual exchange of knowledge and ability to move to the next stage of the growth process. In this perspective, the continuous mother/child/environment relationships, activated by an innate predisposition of the child to social interaction, acquire a determining role, thanks to which a strong sense of self will be created. The feedback that the caregiver gives to the child and the quality of attachment between the child and his parents are fundamental, allowing the child to acquire a healthy mental representation of themselves and others. In this way, the normal psychophysical development of the child takes place, through the building of an identity, managing of new emotional relationships, and through the formation of stable attachment, in order to develop a sense of security and confidence towards of the surrounding world. This delicate growth mechanism, however, can be undermined by the advent of a traumatic moment, such as the diagnosis of a disease. This experience, in fact, can lead to different reactions both in the pediatric patient and in his parents, and break pre-existing balances within the family nucleus. Together with feelings of helplessness in facing the negative situations, anxiety in managing the unknown, and the concern for the future, there are also stressors such as the distancing of the child from their main life context, the considerable changes in his routines, and the perceived menace regarding the clinical situation. The key feature for effective therapy is a strong and mutually confident relationship between teachers and parents, as indicated in many studies [7,20,21]. 

Parents are not merely passive spectators. They have an active role in the educational and formative development of their children, but also they have to ensure a state of psychological well-being for them. Data from personal interviews suggest that teachers in hospitals need to feel part of a multidisciplinary network, including doctors, medical staff, and school-as-institution. A successful Si-Ho program requires the hospital teacher to work closely with the hospital’s medical staff to ensure successful planning and implementation of instruction, as well as fostering a positive and collaborative Si-Ho climate [22].

With regard to this, it is useful to implement the number and frequency of multidisciplinary meetings between teachers and medical staff. Moreover, it is most important to keep a real connection with children’s original school, where they will return after healing in order to ensure the academic continuity for the child. The relation between the original school and the hospital one can be supported by the electronic register and through the creation of a personal portfolio with recorded activities of the student. 

The teacher in the hospital, as described above, should have some special characteristics. In this regard, it would be desirable to prepare broader training programs for this professional role. In particular, this skill set should include management of intense emotional experiences and the management of relationships characterized by suffering. The teaching of communication skills (assertiveness and social skills training) and functional coping strategies (strategies focused on emotions, problems, and coping skills) should also be implemented in this context [23,24]. Another aspect Si-Ho teachers should be trained in relates to the prevention of the infectious risk, through the knowledge of hygienic sanitary norms [25]. In these cases, contact even with opportunistic pathogens—that is, with microorganisms capable of expressing their potential pathogenicity when the host’s immune defenses are compromised—is a credible danger for immunocompromised patients. Therefore, one of the links in the teacher’s training chain is the knowledge of the hygiene and health regulations and the procedures necessary to operate in hospitals, dealing with situations with awareness and responsibility with a view to preventing infectious complications. Conscious behaviors, such as proper hand-washing techniques, avoiding very close contacts, and avoiding contact with Flügge particles which, emitted with speech or coughing, can carry microorganisms, are certainly fundamental tools that the Si-Ho teacher must use to ensure that his didactic-educational intervention does not become a danger for the pediatric student-patient.

In conclusion, the advances made by modern medicine over the last few decades have meant that complex diseases, often with a fatal outcome, can now be well managed, resulting in a high number of pediatric patients who are able to return to school. However, the condition of recovery of health is reached after a sometimes long and tortuous path, often involving trauma to the psychological sphere of the patient. If the subject is a minor, this problem becomes more demanding, as children and adolescents experience the state of the disease in a different way from adults.

It would be convenient for students to look back on their hospital experience without lack or fear, but as a period with its own sense and worth: an enriching experience that adds value to the life story of children and families, as well as to the community, classmates, and to teachers [26]. 

Therefore, it is important to underline that hospitalization is an experience that involves multiple actors, resources, and relational dynamics. The hospitalized child is a child who can be afraid, but also have courage. The parents of a hospitalized child are parents who may experience anxiety and frustration, but at the same time may be a solid reference for their child. The hospital can be a strange and disorienting place, but also be a comfortable and safe place.

Facing this complexity, only by considering the different aspects associated with individual life and development it is possible to understand the peculiarity of each hospitalization and to promote the psychophysical well-being of children and their families. 

## Data Availability

No new data were created or analyzed in this study.
